# Severe Organ Involvement in Systemic Lupus Erythematosus: A Retrospective Single-Center Study From Morocco

**DOI:** 10.7759/cureus.119367

**Published:** 2026-11-09

**Authors:** Lina Lakdim, Abdelhamid Naitlhou, Roseita Carroll

**Affiliations:** 1 Intensive Care Unit, Imperial College Healthcare NHS Trust, London, GBR; 2 Internal Medicine, Cheikh Khalifa University Hospital, Mohammed VI University of Health Sciences, Casablanca, MAR; 3 Cardiac Intensive Care Unit, Imperial College Healthcare NHS Trust, London, GBR

**Keywords:** autoimmune haemolytic anaemia, lupus nephritis, morocco, neuropsychiatric lupus, north africa, retrospective study, systemic lupus erythematosus

## Abstract

Introduction

Systemic lupus erythematosus (SLE) is a chronic autoimmune disease characterized by multisystem inflammatory involvement and an unpredictable clinical course. SLE is among the most prevalent autoimmune diseases in Morocco, yet epidemiological and clinical data from this region remain limited. Disease management relies mainly on conventional therapies, with restricted access to advanced targeted treatments.

Objective

To describe the epidemiological, clinical, biological, and therapeutic characteristics of patients diagnosed with SLE in an internal medicine department in Morocco, with a particular focus on severe renal, hematological, and neurological manifestations.

Methods

We conducted a retrospective descriptive study including 61 patients diagnosed with SLE between January 2017 and September 2022 at the Internal Medicine Department of the International University Hospital Cheikh Khalifa Ibn Zaid in Casablanca, Morocco. Diagnosis was established according to the 2019 European League Against Rheumatism/American College of Rheumatology (EULAR/ACR) and/or the 2012 Systemic Lupus International Collaborating Clinics (SLICC) classification criteria. Data were collected from medical records and analyzed using descriptive statistical methods.

Results

The study population was predominantly female (n=55; 90.2%), with a mean age at diagnosis of 40.3 years (SD 14.2; median 40; range 13-74). The most common manifestations were fatigue (82.0%), arthralgia (70.5%), and malar rash (59.0%). Hematological involvement was prominent: anemia was present in 29 patients (47.5%), of whom 10 (16.4%) had confirmed autoimmune hemolytic anemia (AIHA). Renal involvement was identified in 10 patients (16.4%); among the seven who underwent biopsy, class IV lupus nephritis predominated (n=5; 71.4%). Neurological involvement was documented in 14 patients (22.5%), with the majority comprising nonfocal manifestations such as persistent headaches and mood disorders. Most patients were treated with hydroxychloroquine (n=48; 78.7%) and systemic corticosteroids (n=38; 62.3%); immunomodulatory therapy was required in 18 patients (29.5%). Of the 39 patients with complete follow-up (median 28 months), 14 (35.9%) achieved complete remission, 19 (48.7%) achieved partial remission, 13 (33.3%) experienced disease relapse, and six (15.4%) achieved neither.

Conclusion

This single-center Moroccan cohort highlights a substantial burden of hematological involvement and reveals a treatment landscape defined by restricted access to biologic therapies. The findings underscore the need for region-specific epidemiological data, improved access to targeted treatments, and dedicated prospective research to better characterize SLE in North African populations.

## Introduction

Systemic lupus erythematosus (SLE) is one of the most common systemic autoimmune diseases, predominantly affecting young women. It is characterized by chronic multisystem inflammatory involvement associated with the production of autoantibodies directed mainly against nuclear components. The clinical course is highly variable and unpredictable, typically marked by alternating periods of flare and remission.

The prevalence of SLE varies widely across the world, with higher incidence rates reported in non-Caucasian populations, in whom the disease often presents with more severe clinical manifestations. Globally, prevalence estimates range from 20 to 150 cases per 100,000 individuals [1-3]. In Morocco, SLE represents a public health challenge, accounting for 4.2% of confirmed autoimmune disease diagnoses in a national reference laboratory cohort and ranking as the most common autoimmune disease in that setting [3,4]. The diagnosis of SLE is established using internationally validated classification systems, including the 2019 European League Against Rheumatism/American College of Rheumatology (EULAR/ACR) criteria and the 2012 Systemic Lupus International Collaborating Clinics (SLICC) criteria, both of which demonstrate strong sensitivity and specificity across diverse ethnic populations [5,6]. However, epidemiological and clinical data from Morocco and North Africa remain limited compared with those available from Europe, North America, and Asia.

In addition, the management of SLE relies largely on international recommendations derived primarily from Western and European studies. Access to innovative therapeutic options, particularly targeted biologic therapies, remains restricted in many resource-limited settings. These constraints may influence disease severity, clinical presentation, disease evolution, and long-term prognosis.

The aim of the present study is to describe the epidemiological, clinical, biological, and therapeutic profiles of patients diagnosed with SLE in an internal medicine department in Morocco, with a particular focus on severe renal, hematological, and neurological manifestations.

## Materials and methods

This was a single-center, retrospective, descriptive study conducted between January 2017 and September 2022 in the Internal Medicine Department of the International University Hospital Cheikh Khalifa Ibn Zaid in Casablanca, Morocco.

A total of 87 patient records were initially screened, of which 61 met the inclusion criteria and were included in the final analysis (Figure 1). Patients were included if they had a confirmed diagnosis of SLE established according to the 2019 EULAR/ACR classification criteria and/or the 2012 SLICC criteria [5,6]. Records were considered insufficient and excluded if core diagnostic data, including autoantibody profiles, clinical presentation at diagnosis, or treatment history, were absent or incomplete (n=26 excluded).

**Figure 1:**
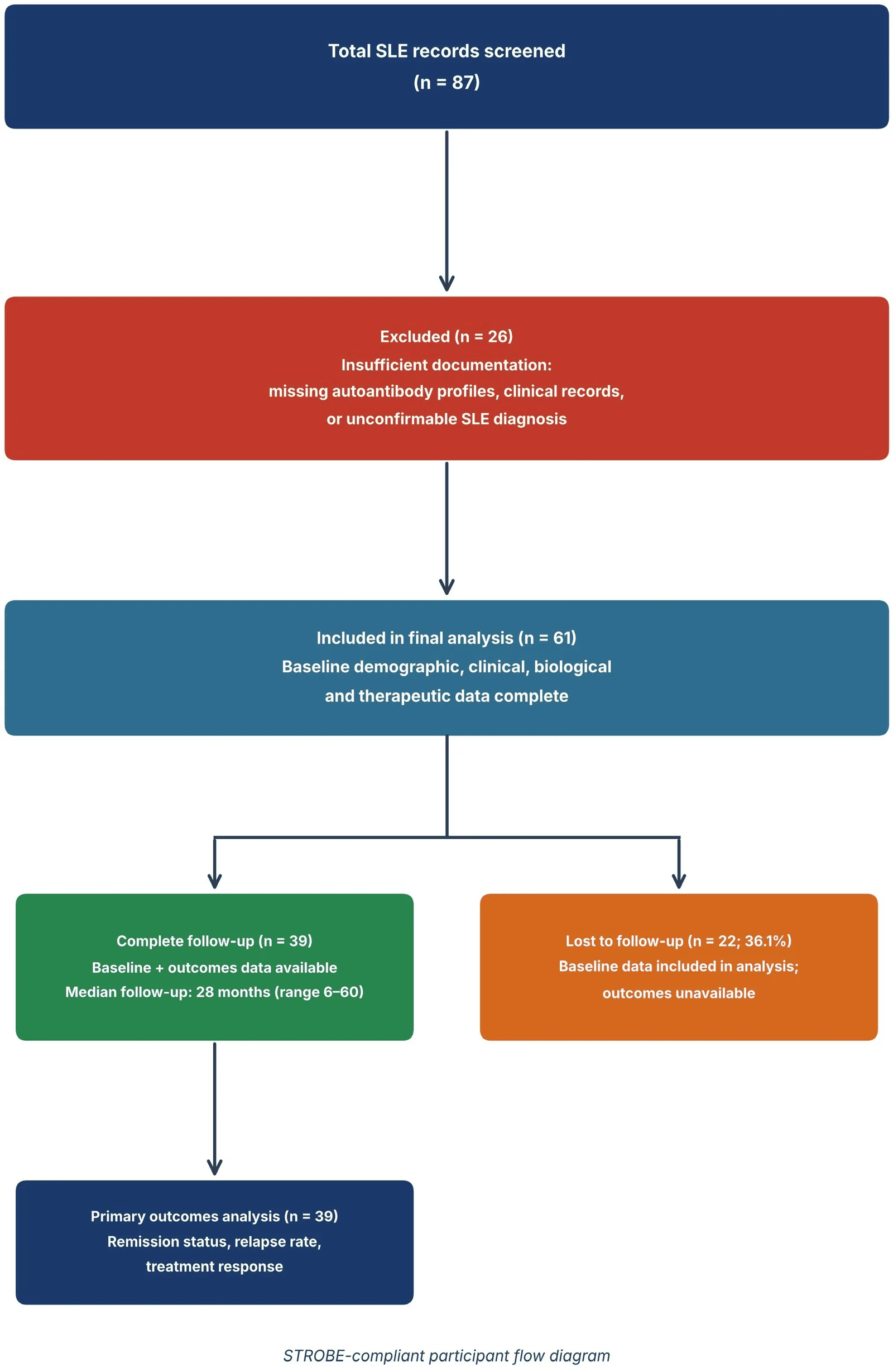
STROBE-compliant participant flow diagram showing the number of records screened (n=87), excluded (n=26), and included in the full analysis (n=61), with a further breakdown of patients with complete follow-up (n=39) and those lost to follow-up (n=22) STROBE: Strengthening the Reporting of Observational Studies in Epidemiology; SLE: systemic lupus erythematosus

The hematological thresholds used were as follows: hemoglobin <12 g/dL for anemia in women and <13 g/dL in men; platelet count <100×10⁹/L for thrombocytopenia; and white blood cell count <4×10⁹/L for leukopenia. Neuropsychiatric manifestations were classified according to the 1999 ACR nomenclature for neuropsychiatric lupus syndromes. Complete remission was defined according to the definition of remission in SLE (DORIS) framework as the absence of clinical and serological disease activity on stable background therapy; partial remission was defined as clinically significant improvement with persistence of mild residual abnormalities [7].

All 61 patients were included in the baseline demographic, clinical, biological, and therapeutic analyses. Of these, 22 patients (36.1%) were subsequently lost to follow-up during the study period, having attended the department for at least one year before discontinuing follow-up. Accordingly, outcome data (remission status and relapse rates) were based on the 39 patients with complete follow-up data.

Demographic, clinical, biological, immunological, and therapeutic data were collected retrospectively using a standardized data collection form. Data analysis was performed using Microsoft Excel (Microsoft Corp., Redmond, WA) and IBM SPSS Statistics (IBM Corp., Armonk, NY). Quantitative variables were expressed as means with standard deviations and medians with ranges; qualitative variables were expressed as absolute frequencies and percentages.

This study was conducted in accordance with the Declaration of Helsinki. Given the retrospective and observational nature of the study, informed consent was waived, and patient data were anonymized.

## Results

Demographic characteristics

A total of 61 patients were included in this study. The population was predominantly female (n=55; 90.2%), with a female-to-male ratio of 8.77:1. The mean age at diagnosis was 40.3 years (standard deviation, 14.2 years; median, 40 years; range, 13-74 years).

General clinical manifestations

Rheumatological and general manifestations were the most frequently observed. Fatigue was the leading systemic symptom, reported in 50 patients (82.0%). Musculoskeletal involvement was present in the majority of the cohort, with arthralgia in 43 patients (70.5%) and polyarthritis in 19 patients (31.1%). Cutaneous manifestations were frequent, including malar rash in 36 patients (59.0%) and photosensitivity in 24 patients (39.3%), along with alopecia in 19 patients (31.1%) and discoid lupus lesions in five patients (8.2%). No cases of generalized rash or other chronic cutaneous lupus erythematosus variants (such as tumid lupus or lupus panniculitis) were identified in this cohort. The full distribution of clinical, hematological, renal, and neurological manifestations is presented in Table 1 and Figure 2.

**Table 1:** Clinical, hematological, renal, and neurological manifestations in patients with SLE (n=61) Some patients presented with more than one clinical manifestation. AIHA: autoimmune hemolytic anemia; ISN: International Society of Nephrology; RPS: Renal Pathology Society; SLE: systemic lupus erythematosus

Clinical manifestation	n	%
Musculoskeletal involvement		
Arthralgia/arthritis	43	70.5
Polyarthritis	19	31.1
General manifestations		
Fatigue	50	82
Fever	9	14.8
Weight loss	13	21.3
Cutaneous involvement		
Malar rash	36	59
Photosensitivity	24	39.3
Alopecia	19	31.1
Discoid lesions	5	8.2
Hematological manifestations		
Any anemia	29	47.5
Inflammatory anemia	19	31.1
AIHA	10	16.4
Lymphopenia	21	34.4
Leukopenia	13	21.3
Thrombocytopenia	6	9.8
Renal involvement		
Renal involvement (overall)	10	16.4
Lupus nephritis class IV (ISN/RPS)	5	8
Lupus nephritis class V (ISN/RPS)	1	1.6
Focal segmental glomerulosclerosis	1	1.6
Neurological manifestations		
Neurological involvement (overall)	14	22.5
Persistent headaches	6	9.6
Mood disorders	4	6.4
Peripheral neuropathy	3	4.8
Cognitive impairment	3	4.8
Epileptic seizures	2	3.2
Ischemic stroke	2	3.2
Acute delirium	2	3.2
Inflammatory polyradiculoneuropathy	1	1.6

**Figure 2:**
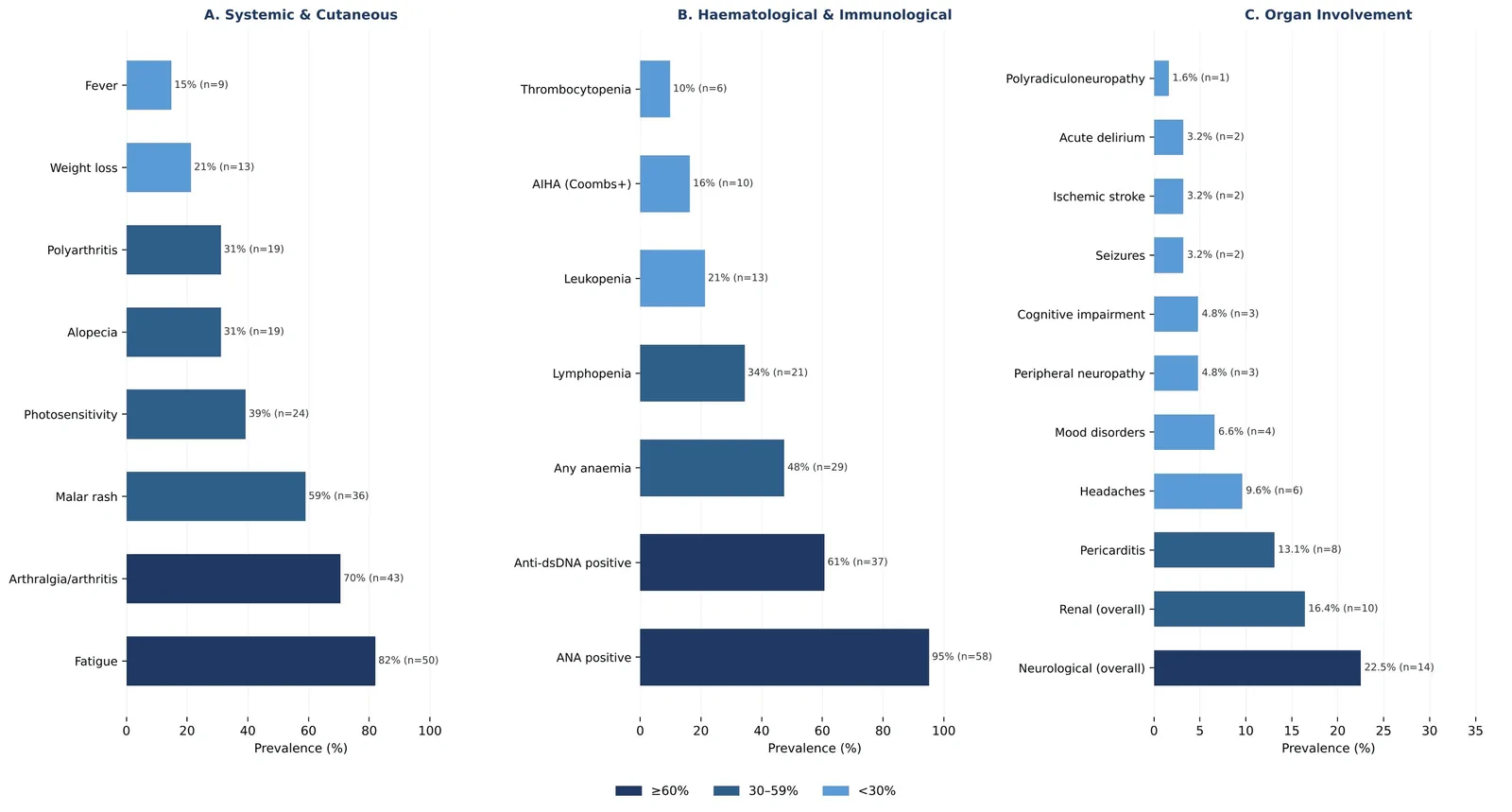
Clinical and biological manifestations across systemic, hematological, and organ-specific domains (n=61) Panel A: systemic and cutaneous manifestations. Panel B: hematological and immunological findings. Color intensity reflects prevalence: dark blue (≥60%), medium blue (30%-59%), and light blue (<30%). Panel C: organ-specific involvement. All values are expressed as percentages, with absolute frequencies, for the total cohort (n=61). AIHA: autoimmune hemolytic anemia; ANA: antinuclear antibodies; dsDNA: double-stranded DNA Created using Python Matplotlib (Python Software Foundation, Wilmington, DE, USA).

Biological, hematological, and immunological findings

Anemia was observed in 29 patients (47.5%) and was predominantly inflammatory, normochromic, and normocytic in nature. Autoimmune hemolytic anemia (AIHA), confirmed by a positive direct Coombs test, was identified in 10 patients (16.4%). This proportion is notably higher than the internationally reported pooled prevalence of AIHA in SLE, estimated at approximately 12.5% in large multi-study meta-analyses, suggesting a more aggressive hematological disease expression in our cohort, although referral bias toward more severe presentations at our tertiary center must also be considered [8].

Other hematologic abnormalities were frequently noted, including lymphopenia in 21 patients (34.4%), leukopenia in 13 patients (21.3%), and thrombocytopenia in six patients (9.8%).

From an immunologic standpoint, antinuclear antibodies (ANA) were positive in 58 patients (95.1%), confirming the autoimmune nature of the disease. Anti-double-stranded DNA (anti-dsDNA) antibodies were detected in 38 patients (62.9%) and were more frequently associated with active disease and visceral involvement. Anti-Sm antibodies were present in a smaller proportion of patients, while antiphospholipid antibodies, including lupus anticoagulant and anticardiolipin antibodies, were identified in a minority of cases; some of these patients presented with thrombotic or neurological manifestations. The complete immunologic profile is illustrated in Figure 3.

**Figure 3:**
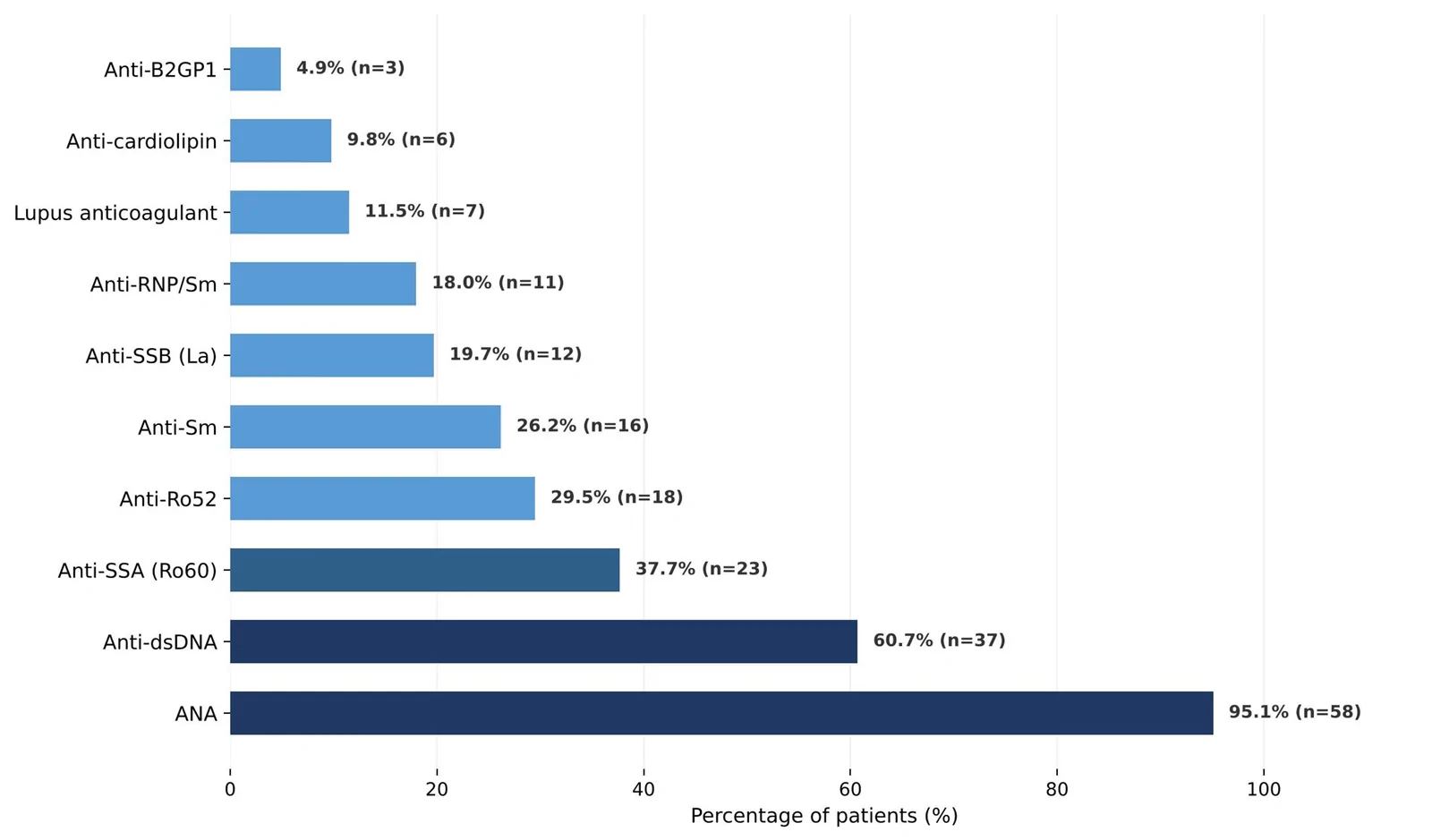
Immunological profile of patients with SLE (n=61) All values are expressed as percentages with absolute frequencies. ANA: antinuclear antibodies; dsDNA: double-stranded DNA; SSA/SSB: Sjögren syndrome antigens; Ro52: Ro52 ribonucleoprotein; Sm: Smith antigen; RNP: ribonucleoprotein; B2GP1: beta-2 glycoprotein 1; SLE: systemic lupus erythematosus Created using Python Matplotlib (Python Software Foundation, Wilmington, DE, USA).

Renal involvement

Renal involvement was observed in 10 patients (16.4%), which is lower than the 40%-60% reported in large international cohorts [9,10]. Renal biopsy was performed in seven of the 10 patients with renal involvement (70.0%). In the remaining three patients, biopsy was not performed due to a combination of patient refusal and financial constraints, as renal biopsy carries significant out-of-pocket costs in Morocco and is not universally covered by the public health system. According to the International Society of Nephrology/Renal Pathology Society (ISN/RPS) classification, class IV lupus nephritis was predominant among biopsied cases, accounting for five patients (71.4%), reflecting diffuse and severe renal involvement with a high risk of progression [9]. One case of class V lupus nephritis and one case of focal segmental glomerulosclerosis were also identified. Importantly, the predominance of class IV nephritis among those who could afford biopsy suggests that the true burden of severe renal disease in our cohort may be underestimated, as patients with milder degrees of involvement may have been less likely to proceed to histological confirmation.

Neurological manifestations

Neurological involvement was observed in 14 patients (22.5%). It is important to note that the majority of these cases comprised nonfocal manifestations: persistent headaches were reported in six patients (9.6%) and mood disorders in four patients (6.4%). A smaller subset represented severe attributable neuropsychiatric SLE (NPSLE): epileptic seizures occurred in two patients (3.2%), ischemic stroke in two patients (3.2%), peripheral neuropathy in three patients (4.8%), acute delirium in two patients (3.2%), and inflammatory polyradiculoneuropathy in one patient (1.6%). Neuropsychiatric manifestations were classified according to the 1999 ACR nomenclature. The rate of 22.5% is consistent with international estimates of severe attributable NPSLE (~20%-25% of SLE patients), as the broader 30%-40% figure reported in large international series includes nonfocal manifestations.

Management and disease evolution

Treatment decisions at our center were guided by disease severity and, critically, by the affordability and accessibility of therapeutic options. In the absence of a formalized institutional treatment protocol, management was largely individualized. Hydroxychloroquine and systemic corticosteroids, being the most affordable and widely available agents in our setting, formed the therapeutic backbone for the majority of patients. Of the 61 patients, 43 (70.5%) were managed with hydroxychloroquine and/or corticosteroids as their primary and only therapeutic approach, as their disease profile, predominantly systemic symptoms, musculoskeletal involvement, and cutaneous manifestations, did not require immunomodulatory escalation.

Hydroxychloroquine was used in 48 patients (78.7%) and systemic corticosteroids in 38 patients (62.3%), with methylprednisolone pulse therapy administered in seven patients (11.5%). Immunomodulatory therapies were introduced in 18 patients (29.5%), reserved for those with severe or refractory organ involvement. Cyclophosphamide was used in 10 patients (16.4%), predominantly for proliferative lupus nephritis and severe neurological involvement, where its efficacy for induction therapy is well established according to EULAR guidelines [11]. The choice of cyclophosphamide over mycophenolate mofetil (MMF) as the more frequent induction agent in our cohort also reflects relative cost and availability considerations: cyclophosphamide is more widely accessible in the Moroccan public health system. MMF was used in eight patients (13.1%). Azathioprine was used in six patients (9.8%) as maintenance therapy following induction, not as a primary induction agent. A single patient (1.6%) received rituximab for refractory thrombocytopenia. Treatment complications were observed in five patients (8.2%).

Among the 39 patients with complete follow-up data (median follow-up: 28 months; range: 6-60 months), outcomes were as follows. Complete remission, defined according to the DORIS framework as full clinical and serological resolution on stable background therapy, was achieved in 14 patients (35.9%). Partial remission, defined as significant clinical improvement with persistence of mild residual abnormalities, was achieved in 19 patients (48.7%), of whom 13 experienced at least one severe relapse requiring therapeutic escalation during the follow-up period. Six patients (15.4%) achieved neither complete nor partial remission and remained on ongoing therapy for refractory or partially controlled disease. No deaths were recorded over the study period. The remaining 22 patients (36.1%) were lost to follow-up, having attended the department for at least one year before discontinuing contact. This most likely reflects financial barriers, relocation, or a perceived sense of stability rather than disease progression, although this cannot be confirmed. Outcomes and remission status are summarized in Figure 4.

**Figure 4:**
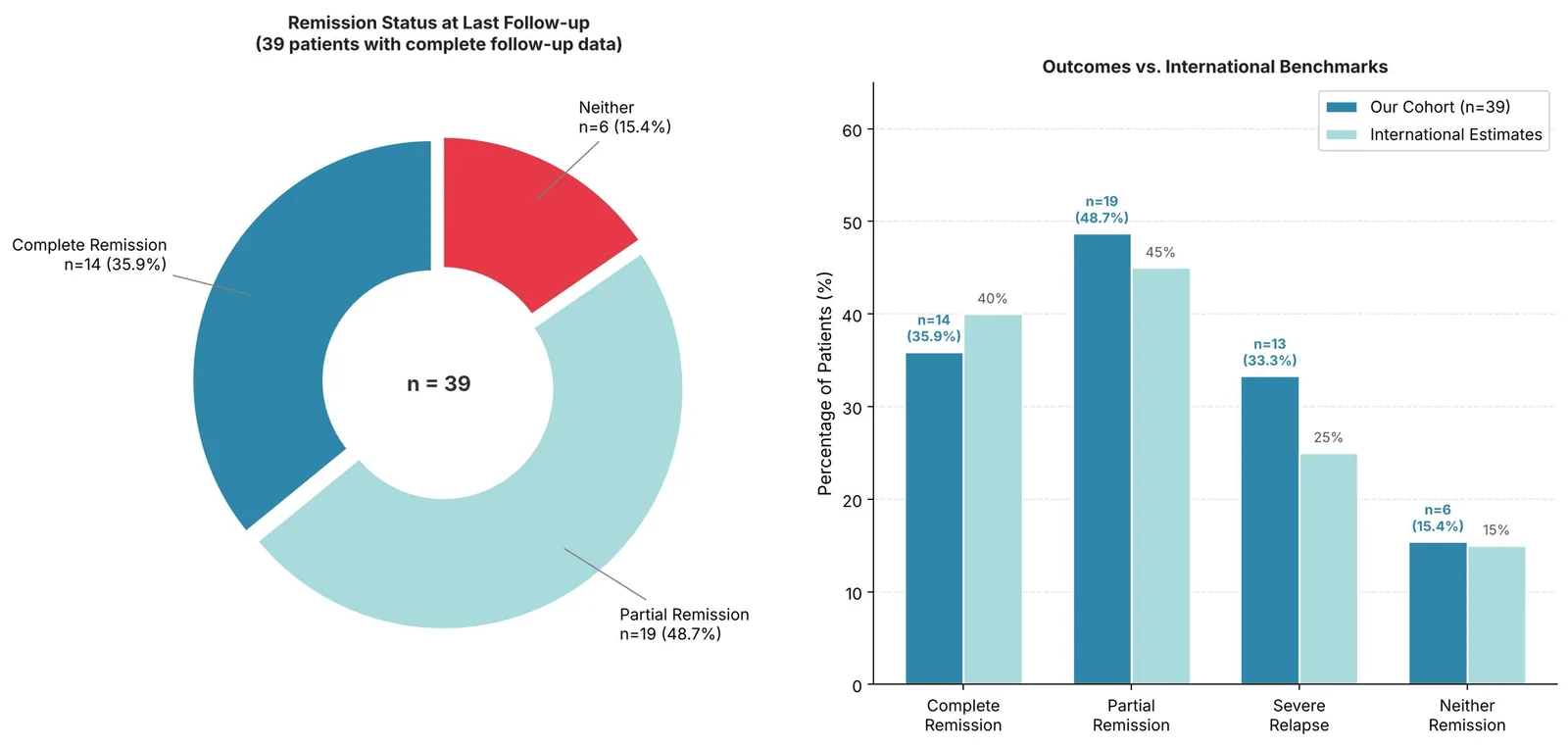
Clinical outcomes and remission status during follow-up (n=39) Complete remission: Full clinical and serological resolution without active organ involvement (DORIS framework). Partial remission: Significant clinical improvement with persistence of mild residual abnormalities. Outcomes were calculated for 39 patients with complete follow-up data; 22 patients (36.1%) were lost to follow-up. International benchmarks were derived from published long-term SLE cohort data. DORIS: definition of remission in SLE; SLE: systemic lupus erythematosus Created using Python Matplotlib (Python Software Foundation, Wilmington, DE, USA).

## Discussion

This study provides clinical data on SLE in a North African population that remains underrepresented in the medical literature. The demographic characteristics of our cohort, particularly the strong female predominance and mean age at diagnosis, are consistent with international data, confirming the universal epidemiological profile of the disease [1-3]. All patients were classified according to the 2019 EULAR/ACR and/or 2012 SLICC criteria, which are widely validated across diverse ethnic populations [5,6].

From a hematological perspective, the frequency of AIHA in our cohort (10 patients; 16.4%) was higher than the internationally pooled prevalence of approximately 12.5% reported in recent meta-analyses [8]. AIHA is a well-recognized marker of active and advanced lupus and has been associated with increased disease severity and poorer outcomes. Its elevated prevalence in our population may suggest a more aggressive hematological disease expression; however, referral bias must be acknowledged as a contributing factor. As a tertiary internal medicine department, our center preferentially attracts patients with acute, severe presentations, including hematological crises, which may overrepresent this manifestation relative to the general SLE population in Morocco.

Although the overall proportion of renal involvement in our series (10 patients; 16.4%) was lower than the 40%-60% reported in large international cohorts, this discrepancy likely reflects a combination of factors [9,10]. First, patients with predominant or severe renal disease may be referred directly to nephrology services rather than remaining under internal medicine follow-up, meaning our cohort may not capture the full renal burden. Second, the significant financial barriers to renal biopsy in Morocco mean that subclinical or mild renal involvement may go unconfirmed and therefore unrecorded. Third, the absence of systematic urine protein quantification at each visit may have led to underdetection of early nephritis. Despite the lower overall rate, the severity of disease among confirmed cases was striking: class IV lupus nephritis, which carries a high risk of progression to chronic kidney disease, predominated among biopsied cases, suggesting that the true burden of severe renal disease may be underestimated in our cohort.

Neurological manifestations were observed in 14 patients (22.5%), consistent with international estimates for severe attributable NPSLE (~20%-25%) [12,13]. It is important to note that the majority of these cases in our cohort comprised nonfocal manifestations, persistent headaches (six patients; 9.6%) and mood disorders (four patients; 6.4%), with a smaller subset representing severe attributable NPSLE (seizures, stroke, peripheral neuropathy, delirium, and polyradiculoneuropathy). In Western cohorts, NPSLE is estimated to affect 30%-40% of patients when using broad ACR case definitions, but the higher end of this range includes nonfocal manifestations that are not directly attributable to SLE [14]. Our rate of 22.5% is therefore broadly comparable. Neuropsychiatric lupus remains one of the most complex aspects of the disease, encompassing a wide spectrum of clinical presentations and being associated with increased morbidity and mortality [14]. The underlying pathophysiological mechanisms are multifactorial and include cerebral vasculitis, thrombotic events related to antiphospholipid syndrome, and the direct neurotoxic effects of autoantibodies [15,16].

In our context, disease management relied mainly on conventional therapies recommended by international guidelines, including hydroxychloroquine, corticosteroids, and immunomodulatory agents for severe organ involvement [11]. However, access to targeted biologic therapies such as belimumab or rituximab remains limited. These treatments have shown promising results in improving disease control and reducing flares, particularly in severe and refractory forms of SLE [17,18].

In contrast, SLE management in Western healthcare systems has increasingly incorporated biologic therapies earlier in the treatment pathway. In European and North American cohorts, belimumab is now approved and used across a broad range of SLE phenotypes [17]. In our series, the complete remission rate of 35.9% (14 of 39 patients) and the partial remission rate of 48.7% compare reasonably with international long-term cohort data, in which complete remission is reported in approximately 19%-27% of patients, although our figures may be modestly inflated by the loss to follow-up of patients with poorly controlled disease. A severe relapse occurred in 33.3% of followed patients (n=13), a finding consistent with the relapsing-remitting course observed in approximately 70% of patients with SLE internationally [19]. Real-world data demonstrate that biologics are used alongside or following conventional immunosuppressants in a substantial proportion of patients with moderate-to-severe disease in high-income settings [20]. Earlier initiation of belimumab has been associated with lower flare rates and delayed organ damage accrual [21]. Combination strategies, such as belimumab following rituximab, have shown significant reductions in anti-dsDNA antibody titers and severe flare rates in randomized controlled trials [22]. In many low- and middle-income countries, including Morocco, biologic therapies for SLE are either unavailable through the public health system or accessible only to patients with private insurance or sufficient financial resources [23,24]. These findings are illustrated in Figure 5.

**Figure 5:**
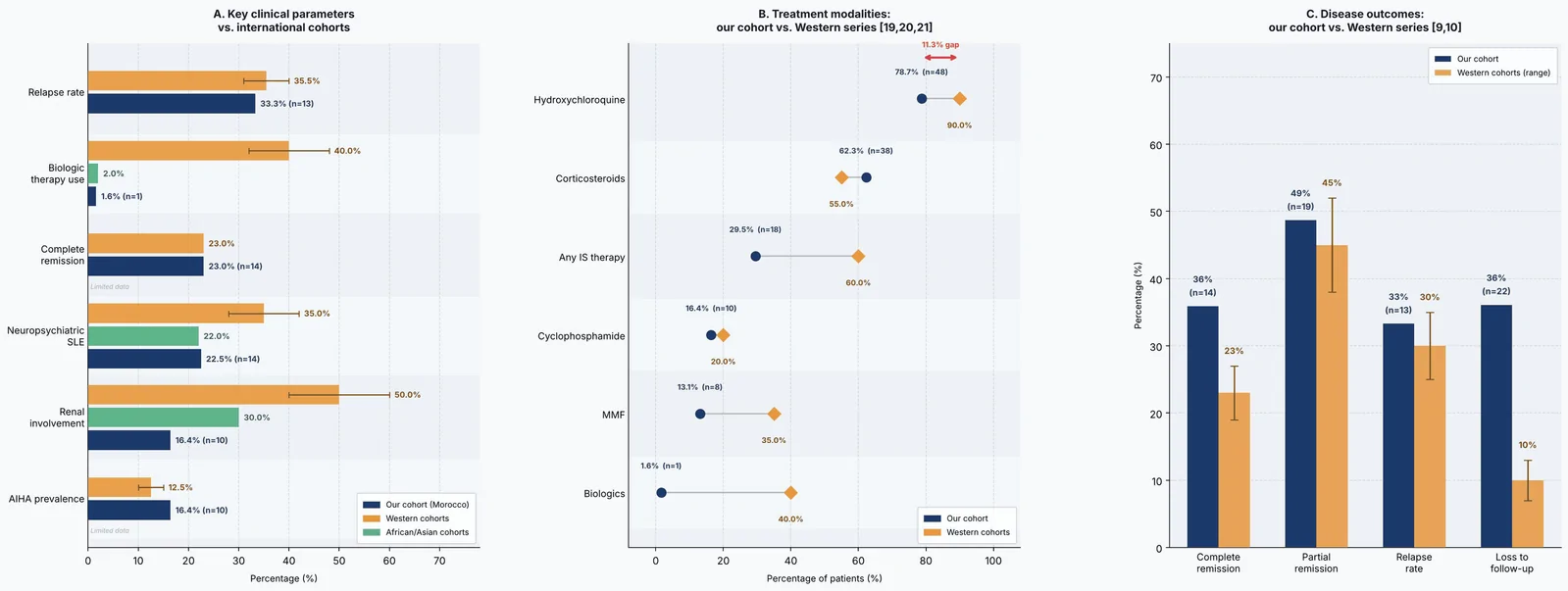
Comparison of key clinical parameters, treatment modalities, and disease outcomes between our Moroccan cohort and international series Panel A: clinical parameters vs. Western and African/Asian cohorts. Panel B: treatment modalities vs. Western series. Panel C: disease outcomes vs. Western series. IS: immunosuppressive; AIHA: autoimmune hemolytic anemia; MMF: mycophenolate mofetil; SLE: systemic lupus erythematosus Created using Python Matplotlib (Python Software Foundation, Wilmington, DE, USA).

Beyond treatment access, the differences in disease expression observed between our cohort and Western series may reflect a complex interplay of genetic and environmental factors. From a genetic standpoint, SLE susceptibility varies significantly across ancestral groups, with non-European populations demonstrating a two- to fourfold higher prevalence and a higher risk of severe organ involvement [25]. Large trans-ancestral genome-wide association studies have identified both shared and ancestry-specific susceptibility loci, with the human leukocyte antigen (HLA) region showing the greatest inter-ancestral divergence. The North African population is genetically distinctive, representing an admixture of indigenous Amazigh (Berber), Arab, sub-Saharan African, and Mediterranean European ancestries, a profile not yet systematically characterized in SLE genetic studies [26]. The first SLE genome-wide association study (GWAS) conducted in an Egyptian population identified novel susceptibility loci not previously reported in European or Asian cohorts, suggesting distinct immunogenetic pathways in this population [26-28]. Furthermore, autoantibody profiles, including higher anti-dsDNA and anti-Sm positivity, have been documented at greater frequencies in patients of African ancestry [27]. The genetic heterogeneity of Arab and North African populations, as inferred from HLA gene distributions, further supports the notion of ancestry-specific disease pathways [29].

From an environmental perspective, Morocco's climate is characterized by high levels of ultraviolet (UV) radiation, particularly in inland and southern regions. UV exposure is a well-established trigger of SLE flares and photosensitivity and may contribute to earlier disease onset and more frequent cutaneous and systemic exacerbations [30]. Paradoxically, SLE patients who practice photoprotection, as recommended, may develop relative vitamin D deficiency, which has itself been associated with increased disease activity and poorer outcomes [31]. Additionally, socioeconomic factors, including delayed diagnosis, reduced access to specialist care, and lower rates of therapeutic monitoring, may compound these biological vulnerabilities. Taken together, these considerations suggest that the severity profile observed in our cohort is likely multifactorial, shaped by ancestral genetic architecture, environmental exposures, and systemic inequities in healthcare access, all of which warrant dedicated prospective investigation.

Limitations

This study has several limitations. First, the retrospective, single-center design introduces inherent selection bias and limits the generalizability of the findings beyond our institution. Second, as a tertiary internal medicine department, our center is likely to attract patients with more severe and complex presentations, introducing referral bias that may overrepresent hematological and other severe manifestations relative to the general SLE population in Morocco. Third, the sample size of 61 patients, while representative of the available caseload at our institution over the study period, remains relatively modest and precludes robust subgroup analyses or multivariable regression. Fourth, 22 patients (36.1%) were lost to follow-up over the study period; outcome figures are therefore based on the 39 patients with complete follow-up data, which may introduce attrition bias and lead to underestimation of the true relapse burden. Fifth, the absence of standardized disease activity scoring using the SLE Disease Activity Index (SLEDAI) at each visit and the lack of damage assessment using the SLICC/ACR Damage Index (SDI) limit the depth of longitudinal outcome analysis. Finally, the study period predates the broader availability of newer targeted therapies, meaning that the therapeutic landscape described here may evolve as access improves. Despite these limitations, this study provides rare and clinically relevant regional data from a North African population that remains systematically underrepresented in the global SLE literature.

## Conclusions

SLE remains a frequent and potentially severe disease in Morocco. This study confirms the characteristic female predominance and young age at onset, while highlighting a substantial burden of hematological involvement and a disease management landscape constrained by restricted access to biologic therapies. The elevated rate of AIHA and the predominance of class IV lupus nephritis among biopsied cases point to clinically severe disease among those reaching tertiary care, although referral bias and barriers to renal biopsy must be considered when interpreting these findings. Treatment in our cohort relied almost entirely on hydroxychloroquine, corticosteroids, and conventional immunosuppressants, while biologic therapies, now integral to SLE management in high-income settings, remain largely inaccessible.

Bridging this treatment gap, alongside investment in region-specific prospective research and genetic epidemiology, represents both a clinical imperative and a health equity issue for North African patients living with SLE.
